# Relationship between cognitive disengagement syndrome and headache in children: insights from biochemical markers

**DOI:** 10.3389/fpsyt.2025.1727744

**Published:** 2025-12-29

**Authors:** Öznur Adıgüzel Akman, Nihal Yıldız

**Affiliations:** 1 Department of Child and Adolescent Psychiatry, Zonguldak Bulent Ecevit University, Zonguldak, Türkiye; 2 Department of Pediatric Neurology, Zonguldak Bulent Ecevit University, Zonguldak, Türkiye

**Keywords:** B12, cognitive disengagement syndrome, ferritin, fT4, headache, TSH, vitamin D

## Abstract

**Objective:**

This study aims to explore the possible relationship between Cognitive Disengagement Syndrome (CDS) and headaches in children and to understand the biochemical basis of this association.

**Materials and methods:**

The study included children aged 6–16 who presented with headaches to the pediatric neurology and child psychiatry outpatient clinics of Zonguldak Bülent Ecevit University Medical Faculty. A total of 42 children with headaches and 40 healthy controls were enrolled. All participants completed a sociodemographic data form, the Barkley Child Attention Scale (BCAS), and the DSM-5-based Atilla Turgay ADHD Rating Scale. In addition, laboratory evaluations included complete blood count parameters, ferritin, vitamin D, vitamin B12, TSH, and free T4 (fT4) levels.

**Results:**

Of the participants, 51.2% (n=42) were male and 48.8% (n=40) were female. Signs of Cognitive Disengagement Syndrome were found to be significantly higher in children with headache (p<.001). Significant differences were found between children with and without headaches in terms of biochemical parameters, including RBC, hemoglobin, ferritin, vitamin B12, vitamin D, free T4, and TSH levels. There was a negative correlation between BCAS scores and levels of vitamin B12, ferritin, vitamin D, sT4, and TSH (respectively, r = –0.246, p = 0.026; r = –0.361, p = 0.001; r = –0.436, p <0.001; r = –0.382, p <0.001; r = –0.308, p = 0.005).

**Conclusion:**

Childhood headaches may be associated with symptoms of Cognitive Disengagement Syndrome. Our data indicated that decreased levels of vitamin B12, ferritin, vitamin D, sT4, and TSH were associated with higher CDS scores, suggesting a possible link. The potential for CDS development in children with headaches should be considered, and the importance of biochemical parameters in this process should not be overlooked.

## Introduction

Cognitive Disengagement Syndrome (CDS), formerly known as Sluggish Cognitive Tempo (SCT), is characterized by slow cognitive processing, mental sluggishness, daydreaming, reduced motivation, and attention difficulties (1). Although research interest in CDS has increased in recent years, its neurobiological mechanisms remain insufficiently understood. Several studies have investigated biochemical correlates of CDS and have reported potential associations between CDS symptoms and deficiencies in vitamin B12, iron, and vitamin D (2–5). These biochemical markers play essential roles in neurological development, attention, processing speed, and cognitive functioning, suggesting that metabolic alterations may contribute to the cognitive slowing characteristic of CDS.

Headache, on the other hand, is the most common neurological symptom in childhood and is among the most frequent causes of pain. The prevalence of headaches among children (ages 8–12) and adolescents (ages 13–17) varies significantly depending on environmental factors, methodologies used, and diagnostic criteria. Migraine prevalence ranges from 3% to 11%, while tension-type headache prevalence ranges between 0.9% and 24% (6). Most pediatric headaches are primary headaches, consisting mainly of migraines and tension-type headaches (7). Cognitive impairments have also been documented in children and adults with migraine, including difficulties in executive function, attention, processing speed, and memory (8–10). Additionally, biochemical parameters such as ferritin, vitamin B12, and vitamin D have been implicated in the development and severity of headaches, with deficiencies reported more frequently among individuals experiencing recurrent headaches (11–14).

When the existing literature is considered together, it appears that CDS symptoms may be more commonly observed in children presenting with headaches, potentially due to shared biochemical mechanisms. Vitamin B12, ferritin, vitamin D, free T4, and TSH levels have demonstrated significant associations with attention, processing speed, cognitive tempo, and overall neurocognitive function (15–19). However, despite the growing body of research linking biochemical parameters to both CDS and pediatric headaches separately, no study to date has simultaneously examined the relationship between biochemical markers, headache characteristics, and CDS symptoms.

This study aims to address this gap by exploring the potential relationship between headaches and CDS in children and by examining the biochemical basis underlying this association. By analyzing vitamin B12, complete blood count (CBC), ferritin, folic acid, vitamin D, TSH, and free T4 (sT4) levels in children presenting with headaches, this research seeks to identify biochemical factors shared by both conditions and to contribute to a clearer understanding of the neurobiological mechanisms connecting CDS and pediatric headaches.

## Materials and methods

This study was conducted on children aged 6–16 who presented with headaches to the pediatric neurology and child psychiatry clinics of Zonguldak Bülent Ecevit University Faculty of Medicine. A total of 42 children with primary headaches and 40 healthy controls were included in the study. All participants completed a sociodemographic data form, the Barkley Child Attention Survey, and the DSM-5-based Atilla Turgay ADHD Rating Scale. Additionally, laboratory evaluations were conducted to assess hemogram parameters, ferritin, vitamin D, vitamin B12, TSH, and free T4 (sT4) levels.

A power analysis was conducted using G*Power (version 3.1) to determine the required sample size. Assuming a medium effect size (d = 0.5) for differences in biochemical parameters and CDS scores between groups, a significance level of α = 0.05, and a power of 0.80, the minimum required sample size was calculated as 64 participants (32 per group). Our final sample of 82 children (42 with headaches, 40 controls) exceeded this requirement.

IRB approval for the study was procured from the Ethics Committee of Zonguldak Bülent Ecevit University [2024/22]. All of the study procedures were in accordance with the WHO Declaration of Helsinki and local laws and regulations.

### Data collection tools

#### Sociodemographic data form

The semi-structured form prepared by the researchers includes information about the participants’ age, date of birth, number of siblings, grade level, whether they have any health problems, whether they have previously consulted a child psychiatrist, parents’ educational level, monthly income, and any family history of psychiatric illness. It also consists of questions regarding headaches, including their frequency, duration, and timing.

#### Diagnostic evaluation of headaches

All children in the headache group were evaluated face-to-face by a pediatric neurologist. Headache diagnoses were established using the International Classification of Headache Disorders, 3rd edition (ICHD-3). Primary headaches were categorized as migraine or tension-type headache based on the ICHD-3 criteria, which include headache duration, location, quality, associated symptoms (e.g., photophobia, phonophobia, nausea), and impact on daily functioning.

Headache severity was assessed using a 10-cm Visual Analog Scale (VAS), on which children (with parental support when necessary) rated their average headache intensity during the previous month. Headache frequency, duration, and timing were recorded via a structured clinical interview.

### The Barkley Child Attention Scale

Created by Russell Barkley in 2013, this questionnaire consists of 12 items. In addition to these items, there are two additional questions that query the age of onset of symptoms and the areas in which impairment in functioning occurs. Developed to assess cases of sluggish cognitive tempo, this scale utilizes a 4-point Likert scale. A cutoff score of 23 has been recommended, and the scale has been validated for Turkish validity and reliability (20, 21).

### DSM-IV Based Screening and Assessment Scale for Disruptive Behavior Disorders- parent form

Developed by Atilla Turgay in 1995 based on DSM-IV diagnostic criteria, this scale aims to assess the presence of ADHD and behavioral problems in children and adolescents. The scale consists of a total of 41 items: 9 items related to inattention, 9 items related to hyperactivity and impulsivity, 8 items related to oppositional defiant disorder, and 15 items related to conduct disorder symptoms. Each item is scored from 0 to 3 based on the clinical condition of the patient. If 6 out of the 9 items related to inattention and hyperactivity-impulsivity receive scores of 2 or 3, diagnostic criteria are considered met (22). The Turkish validity and reliability of this test were established by Ercan et al. (23).

### Measurement of biochemical parameters

Samples were collected in the morning before breakfast. 5 mL venous blood was placed in gel-barrier tubes under sterile conditions. After separation from the serum, the samples were stored at −20 °C in the biochemistry laboratory of the university until analysis. In our study, the serum levels of vitamins were measured by an enzyme-linked immunosorbent assay (ELISA). Ferritin and thyroid hormone levels were measured using standard automated immunoassay systems.

### Statistical analysis

The statistical analyses of the study will be conducted using the Statistical Package for the Social Sciences (SPSS-26.0) for Windows. Descriptive statistics for categorical variables will be presented as frequencies and percentages; for continuous variables, they will be presented as mean, standard deviation, median, minimum, and maximum values. The normality of distribution for continuous variables will be assessed using the Shapiro-Wilk test. For comparisons between two independent groups, the independent samples t-test will be used for normally distributed continuous variables, while the Mann-Whitney U test will be used for non-normally distributed variables. For comparisons of categorical variables between groups, Pearson’s chi-square test, Yates’ correction, Fisher’s exact test, and the Fisher-Freeman-Halton test will be employed. Correlations between continuous variables will be interpreted using Spearman’s and Pearson’s correlation coefficients. In all statistical analyses, a p-value of less than 0.05 will be considered statistically significant.

## Results

A total of 82 children participated in the study, including 42 with headaches and 40 healthy controls. The mean age of the participants was 10.9 ± 3.05 years; 48.8% were female and 51.2% were male. Sociodemographic data and scale scores of the participants are presented in **Table 1**.

**Table 1:** Sociodemographic characteristics and scale scores (n = 82).

		n	%	Age Mean ± SD	BCAS scores ± SD	DBSAS- DBD-Inattentive score ± SD	DBSAS- DBD-Hyperactive score ± SD
Group	patient	42	51,2	12,1 ± 3,34	19,81 ± 7,151	8,52 ± 7,123	6,02 ± 5,97
	control	40	48,8	9,75 ± 2,18	13,08 ± 3,91	11,03 ± 3,60	10,00 ± 21,79
	total	82	100	10,9 ± 3,05	16,52 ± 6,68	9,74 ± 5,79	7,96 ± 5,70
Gender	male	42	51,2	10,81 ± 2,77	14,90 ± 6,15		
	female	40	48,8	11,1 ± 3,35	18,23 ± 6,87		
Education level	primary school middle school	33 29	40,3 35,4				
	high school	20	24,4				

SD, Standard Deviation, BCAS, The Barkley Child Attention Scale; DBSAS- DBD, DSM-IV Based Screening and Assessment Scale for Disruptive Behavior Disorders- Parent form.

Among the biochemical measurements obtained from the participants, levels of WBC, RBC, Hemoglobin, Ferritin, B12, Vitamin D, sT4, and TSH were evaluated. Significant differences were observed between children with and without headaches in biochemical parameters, including RBC, Hemoglobin, Ferritin, B12, Vitamin D, T4, and TSH levels.

The B12 level in children without headaches was found to be approximately 200 pg/mL higher on average compared to children with headaches. This difference was statistically significant (p <0.001). The comparison of biochemical parameters between the groups is presented in **Table 2**.

**Table 2:** Comparison of biochemical parameters in children with headache and healthy controls.

Biochemical parameters	Patient M (SD)	Control M (SD)	p
WBC	8.92 (1.82)	8.67 (2.01)	.435
**RBC**	4.35 (0.45)	4.80 (0.38)	<.001^*^
**Hemoglobin (g/dl)**	11.7 (1.6)	12.7 (0.8)	.001^*^
**Ferritin (ng/mL)**	21.2 (16.7)	46.7 (36.4)	.001^*^
**B12 (pg/mL)**	326.73 (191.11)	530.8 (207.2)	<.001^*^
**Vitamin D (ng/mL)**	15.4 (6.8)	25.0 (7.5)	<.001^*^
**sT4 (µg/dL)**	1.03 (0.24)	1.35 (0.13)	<.001^*^
**TSH (µIU/mL)**	1.83 (0.84)	2.45 (1.01)	.004^*^

M, Mean; SD, Standard Deviation; p <.05 was considered significant.

*Mann-Whitney U test was applied.

WBC, White Blood Cell; RBC, Red Blood Cell; B12, Vitamin B12; sT4, Serum Thyroxine (T4); TSH, Thyroid Stimulating Hormone

Bold values indicate statistically significant differences (p < 0.05)

**Figure 1:**
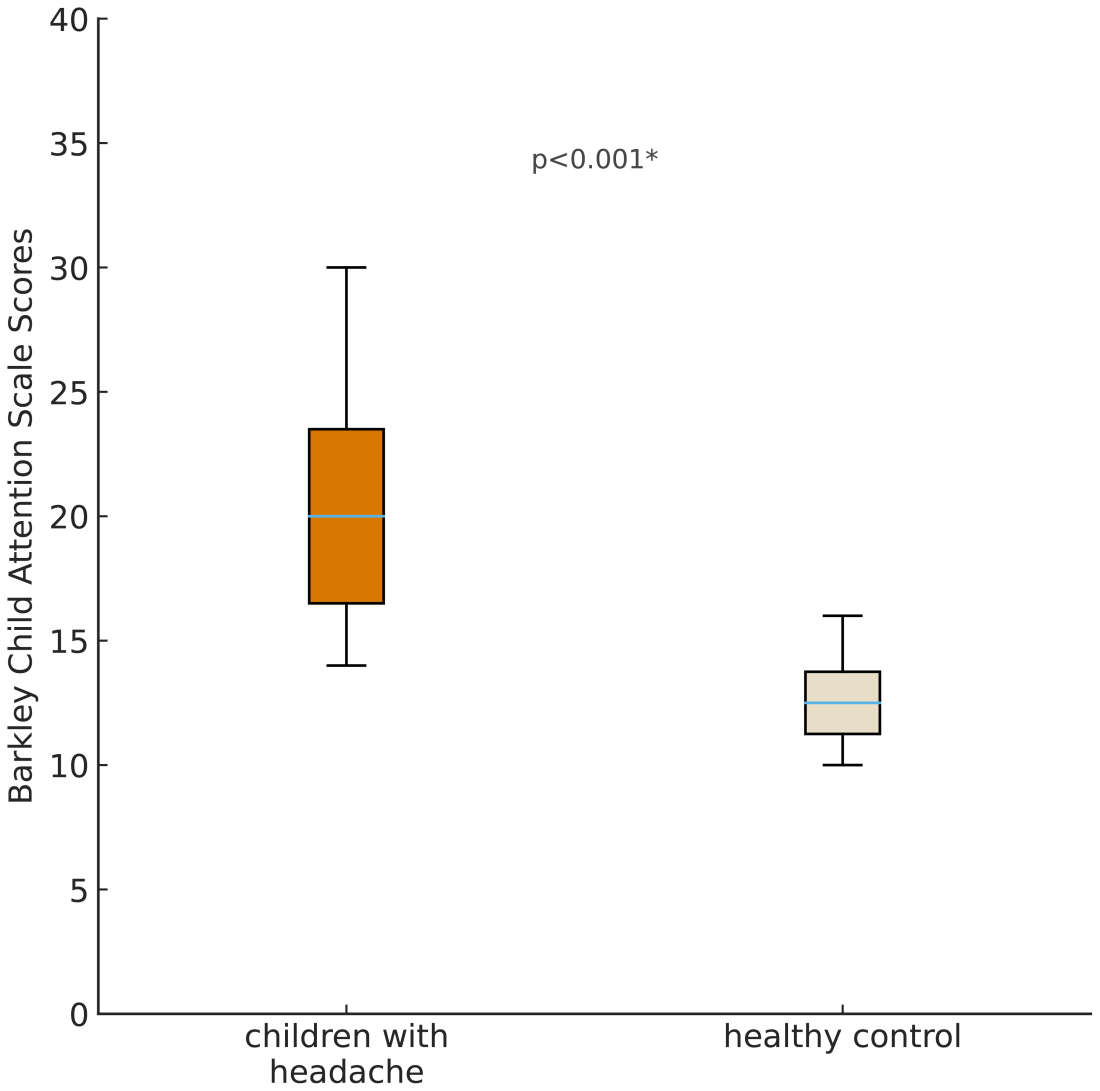
Comparison of Barkley Attention Scale Scores between children with headache and healthy controls.

Children with headaches had higher scores on the BCAS, indicating slower cognitive tempo compared to those without headaches, and this difference was found to be statistically significant (p <0.001). BCAS scores for each group are presented in **Figure 1**.

When examining the correlations between BCAS scores and blood parameters, negative correlations were found with B12, Ferritin, Vitamin D, sT4, and TSH levels. The correlation between blood parameters is presented in **Table 3**.

**Table 3:** Correlations between participants’ BCAS scores and biochemical parameters.

Biochemical parameters	Spearman's r	p
WBC	.120	.281
Hemoglobin (g/dl)	–.202	.069
RBC	–.196	.077
**B12 (pg/mL)**	**–.246**	.026*
**Ferritin (ng/mL)**	**–.361**	.001*
**Vitamin D (ng/mL)**	**–.436**	< .001*
**sT4 (µg/dL)**	**–.382**	< .001*
**TSH (µIU/mL)**	**–.308**	0.005*

Spearman's rho korelasyon katsayıları (r) ve anlamlılık düzeyleri (p)

* p<0.05 was considered statistically significant.

WBC, White Blood Cell; RBC, Red Blood Cell; B12, Vitamin B12; sT4, Serum Thyroxine (T4); TSH, Thyroid Stimulating Hormone; BCAS, Barkley Child Attention Scale

Bold values indicate statistically significant differences (p < 0.05)

## Discussion

In this study, the relationship between headaches, which frequently occur during childhood, and CDS was investigated, along with a potential association between this connection and certain biochemical parameters. Our findings revealed that children experiencing headaches exhibited more CDS symptoms. Furthermore, levels of vitamin B12, ferritin, vitamin D, T4, and TSH were found to be potential determinants in this relationship.

In the literature, both headaches and CDS have been associated with attention deficits, sleep disturbances, and emotional regulation problems (2, 24, 25). Studies have also shown that changes in blood parameters are linked to headaches (12–14, 26, 27).

In some studies investigating the relationship between vitamin D levels and headaches, it has been found that serum vitamin D levels in children with headaches, including migraine and tension-type headaches, are significantly lower compared to children without headaches (26, 28, 29). It has also been reported that vitamin D levels are associated with the severity of headaches (30). In our study, vitamin D levels were lower in children with headaches than in healthy children; however, no relationship was found between vitamin D levels and headache severity (r =-0.264, p = 0.091).

Even though vitamin D levels were low in our study, this deficiency may not have been sufficient to cause clinically significant differences in headache severity. Additionally, since headache severity was mostly based on the children’s self-reports, this may have introduced some variability in the measurements.

Conditions that disrupt sleep quality can contribute to both headache and CDS. One study reported that lethargy and drowsiness in CDS may be associated with reduced energy production due to low vitamin D levels (2). Similarly, in our study, as vitamin D levels decreased, CDS scores increased. It is known that vitamin D has anti-inflammatory and immunomodulatory effects (31). When vitamin D levels decrease, inflammation may increase, which can contribute to headaches. Cognitive slowing associated with headaches may, in turn, exacerbate CDS symptoms. Additionally, vitamin D has been reported to enhance dopamine expression (32), and decreased dopamine levels have been linked to migraine attacks (32). However, given the uniformly low vitamin D levels in our sample, no definitive relationship can be inferred between vitamin D, headaches, or CDS; findings should therefore be considered preliminary.

Ferritin, one of the blood parameters thought to be associated with headaches, has also been investigated in the literature (12, 13, 33, 34). One study reported that iron-deficiency anemia was associated with chronic daily headaches and that headache severity increased with anemia severity. The same study found that serum iron, ferritin, total iron-binding capacity, and transferrin saturation were associated with chronic daily headaches (13). In our study, ferritin levels were also lower in the group with headaches, and as ferritin levels decreased, CDS symptoms increased. Ferritin deficiency has been reported to disrupt sleep regulation (35). Sleep problems due to ferritin deficiency may predispose individuals to headaches and, along with fatigue, may contribute to cognitive slowing and increased CDS symptoms. However, it should be considered that the hormonal changes occurring in girls who are in the menarche period in our study may influence ferritin levels and, consequently, may affect the study’s findings.

A study conducted on pediatric migraine patients reported low levels of vitamin B12 and folic acid (36). Another study showed that dietary folic acid supplementation in women aged 20–50 could help prevent severe headaches (14). Other studies have pointed to the potential benefits of administering B6, folate, and B12 for the prophylaxis of migraines with aura (37).

One study found elevated homocysteine levels in patients with migraines with aura (38). Supporting this, another study showed that B-complex vitamin supplementation could reduce homocysteine levels and consequently decrease headache severity (39). Although we did not directly measure homocysteine levels in our study, B12 levels were found to be lower in children with headaches compared to healthy peers. One study emphasized the relationship between vitamin B12’s effects on methylation pathways and circadian rhythm and CDS symptoms such as low energy, cognitive slowing, and sleepiness (2). Due to the neurological effects of vitamin B12, increases in both headaches and CDS symptoms may be observed. Our findings support this association.

Another parameter investigated in relation to childhood headaches has been thyroid function tests (40, 41). One study found that levothyroxine treatment was effective in reducing migraine headaches in subclinical hypothyroidism (40). Positive associations between hypothyroidism and both migraine and tension-type headaches have also been reported (41). Another study even proposed that headaches may be a risk factor for developing hypothyroidism (42). In our study, thyroid function values were also found to be lower in children with headaches and were associated with headache severity. Although the relationship between thyroid function and cognitive performance has been demonstrated (43), due to the limited literature on CDS, results remain inconclusive. One study reported elevated TSH levels in CDS. (15), whereas another found no such relationship (2).

In addition to all these, other blood parameters investigated in the context of headaches have also been reported in the literature. In one study, C-reactive protein (CRP), a marker of inflammation, was found to be elevated in children with headaches, including migraines, compared to those without headaches (44). In our study, only white blood cell (WBC) counts were examined as an inflammatory marker, and no significant differences were found between the groups.

Another study found that children with headaches had lower total cholesterol levels and higher levels of brain-derived neurotrophic factor (BDNF) compared to the control group. The same study also explored the relationship between lipid profiles and headaches, reporting that lipid abnormalities may be associated with headaches only in children with obesity (45).

Although our study provides novel findings that have not been previously addressed in the literature, it has several limitations. First, some blood biomarkers previously associated with headaches—such as S100B (46) and BDNF (45)—could not be evaluated due to cost constraints. Similarly, other inflammatory markers, including CRP, lipid profiles, and cholesterol levels, were not assessed. Second, the sample consisted of a limited number of participants recruited from a single center, which restricts the generalizability of the results. Additionally, the use of only the parent-report form in the CDS assessment may have increased the risk of observer bias, as teacher-report forms were not available for all participants. Finally, the inability to perform subgroup analyses based on headache subtypes represents another important limitation of the study.

Given the critical roles of vitamin B12, ferritin, and vitamin D in neurotransmitter synthesis, brain oxygenation, and energy metabolism, it is plausible that deficiencies in these biochemical parameters may contribute to the emergence or severity of CDS-related attentional and motivational difficulties. Although the present study was not designed to establish causal relationships, the findings indicate a possible association between biochemical status and cognitive disengagement in children with headaches, underscoring the need for further investigation. While the correlations between biochemical parameters and CDS scores reached statistical significance, their relatively small effect sizes suggest that these biochemical factors may function as contributory biological influences rather than primary determinants of CDS symptoms.

## Conclusion

The findings of this study suggest a possible association between CDS and specific biochemical parameters in children with headaches. This highlights the importance of evaluating both CDS-related symptoms and relevant biochemical indicators when assessing pediatric patients presenting with headaches. In particular, alterations in vitamin B12, ferritin, vitamin D, free T4, and TSH levels may offer meaningful clues for developing individualized clinical approaches and optimizing symptom management.

The observed relationship between childhood headaches and CDS appears to reflect a potential area of interaction supported by biochemical patterns rather than causality. The results indicate that CDS symptoms may be more frequently observed among children with headaches, emphasizing the potential contribution of biochemical factors to this association. Nevertheless, given the cross-sectional design, these findings should be interpreted as correlational. Future studies with larger sample sizes, longitudinal designs, and multidimensional assessment tools are needed to further clarify the nature and direction of these relationships.

Beyond headache-related outcomes, the present findings point to subtle links between biochemical processes and cognitive functioning, suggesting that such associations may provide valuable clinical perspectives. Incorporating parameters such as vitamin B12, ferritin, and vitamin D into the evaluation of children with headaches and cognitive difficulties may support more comprehensive diagnostic reasoning. Additionally, monitoring CDS symptoms alongside headache follow-up may offer further insight into the clinical profile of these patients.

## Data Availability

The original contributions presented in the study are included in the article/supplementary material. Further inquiries can be directed to the corresponding author.
